# Bioactive Metabolites from the Dusty Seeds of *Gastrodia elata* Bl., Based on Metabolomics and UPLC-Q-TOF-MS Combined with Molecular Network Strategy

**DOI:** 10.3390/plants14060916

**Published:** 2025-03-14

**Authors:** Yanduo Wang, Liwen Zhong, Huiqi Fang, Zhao Liu, Peng Wang, Longfei Li, Lin Chen, Gang Ding

**Affiliations:** 1 State Key Laboratory of Bioactive Substance and Function of Natural Medicines, Institute of Medicinal Plant Development, Chinese Academy of Medical Sciences and Peking Union Medical College, Beijing 100193, China; wangyanduo1115@163.com (Y.W.); s2023009049@student.pumc.edu.cn (L.Z.); klxsxjsw@163.com (H.F.); s2024009050@student.pumc.edu.cn (Z.L.); 2 College of Pharmacy, Hebei University, Baoding 071002, China; 18713067307@163.com (P.W.); lilongfei@hbu.edu.cn (L.L.); 3 Comprehensive Utilization of Edible and Medicinal Plant Resources Engineering Technology Research Center, Zhengzhou Key Laboratory of Synthetic Biology of Natural Products, Huanghe Science and Technology College, Zhengzhou 450006, China; lchenchina@163.com

**Keywords:** orchid seeds, *Gastrodia elata* Bl., bioactive/signal metabolites, metabolomics, UPLC-Q-TOF-MS, growth promotion

## Abstract

Orchids produce tiny, light seeds (dust-like seeds without endosperm) that rely on specific symbiotic fungi for successful germination. Plant roots often release small signaling molecules or bioactive compounds to attract arbuscular mycorrhizal (AM) fungi, promoting fungal growth and hyphal branching. However, until now, no such bioactive or signaling molecules have been identified in orchids that help recruit fungi for seed germination. In this study, we used metabolomics and UPLC-Q-TOF-MS/MS, combined with a molecular network approach, to explore potential bioactive/signaling molecules in the seeds of the achlorophyllous orchid *Gastrodia elata* Bl. Our analysis revealed the presence of amino acids, nucleotides, lipids, organic acids, saccharides, phospholipids, and lignanamides. Specifically, organic acids, saccharides, and lignanamides were shown to promote the growth of *Mycena osmundicola*, a fungus important for seed germination. Additionally, lignanamides inhibited the plant pathogen *Fusarium oxysporum* and exhibited strong antioxidant and anti-inflammatory activities. This is the first systematic identification of bioactive/signaling molecules in *G. elata* Bl. seeds, providing new insights into the symbiotic relationship between orchids and fungi.

## 1. Introduction

The best-known and most frequently mentioned features of orchids are their very small and light seeds (also named dusty seeds), which are generally produced in great numbers. *Cycnoches ventricosum* Bateman var. *chlorochilon* (Klotzsch) P. H. Allen, an extreme example, has the capacity to produce up to 4 million seeds per fruiting pod [1]. Orchid seeds contain some primary metabolites such as free sugars, lipids, and protein confirmed by histological sections and GC-MS analysis of mature orchid seeds, but these unique seeds cannot use the energy materials (sugars and lipids) and need to be infected with orchid mycorrhizal (OM) fungi at some phase in their life cycle to provide nutrition for seed germination and seedling development before photosynthesis process [2,3,4,5,6]. In the arbuscular mycorrhizal (AM) symbiosis, host plants usually can exudate small signal molecules from roots such as strigolactones to recruit and trigger AM fungal hyphal branching and spore germination or other metabolites to promote AM fungal growth, in return, AM fungi produce signal molecules lipo-chitooligosaccharides (LCOs) to initiate the mutualistic symbiotic pathway [7]. Yet, abundant research has demonstrated that OM fungi supply essential nutrients such as C, N, S, and other nutrition to their orchid hosts, similarly to the role of arbuscular mycorrhizal (AM) fungi in symbiosis [8]. Furthermore, orchid seeds may synthesize hormone compounds, including jasmonic acid and abscisic acid, which regulate the growth of symbiotic fungi [9]. While various metabolites have been isolated from orchid tubers and their fungal partners, there is a lack of comprehensive studies on signaling or bioactive molecules in orchid seeds that could be involved in OM fungal symbiosis.

*Gastrodia elata* Bl. is a precious and endangered traditional Chinese medicinal plant belonging to one of the achlorophyllous orchids (full mycoheterotrophy lifestyle without photosynthesis), which cannot be planted artificially. With more than 20 years of exploration of the life cycle of *G. elata* Bl., Xu et al. finally figured out that the unique achlorophyllous orchid needs two fungi (*M. osmundicola* and the *Armillaria mellea*) at different stages to provide nutrition for seed germination and plant growth [10,11,12,13]. *M. osmundicola* first penetrates imbibed seeds through hyphae, which are absorbed as nutrients by the seed cells to support germination. The seeds grow up and form protocorms and small white bulbs at a certain stage, which are then provided by *A. mellea* hypha with nutrition till they become mature bulbs of *G. elata* Bl. Finally, the mature bulbs blossom and bear fruits to start another life cycle [10,11,12,13]. After understanding the life history of *G. elata* Bl., people began to plant *G. elata* Bl. on a large scale in mountain areas in the South-East of China, including Guizhou, Yunnan, Chongqing, Hubei, Shanxi, and other Provinces. Now, NHCPRC has announced that *G. elata* Bl. is one of the medicine and food homology plants, which expands its practical uses in medicine, food, health products. and other fields. The primary metabolites and secondary metabolites of the pseudobulb of *G. elata* Bl. have been extensively investigated [14], whereas no signal molecule or bioactive compounds from pseudobulb or seeds of *G. elata* Bl. were reported to recruit or promote the growth of seed germination fungi *M. osmundicola.*

In this report, amino acids, nucleotides, lipids, organic acids and sugars, and lignanamides together with phospholipids were found in the *G. elata* Bl. seeds based on results of metabolomics and UPLC-Q-TOF-MS combined molecular network strategy. A series of lignanamides (**1**–**12**) including two new analogues were also purified from seeds. Organic acids, saccharides, and lignanamides could promote the growth of *M. osmundicola.* Lignanamides showed an inhibitory effect on *F. oxysporum* and displayed strong antioxidant and anti-inflammatory bioactivities. In this report, the bioactive/signal metabolites discovery from seeds of *G. elata* Bl. were presented based on metabolomic and UPLC-Q-TOF-MS chemical profile results.

## 2. Materials and Methods

### 2.1. General Experimental Procedures

The FTIR-8400S spectrophotometer (Shimadzu, Kyoto, Japan) and the J-815 spectropolarimeter (JASCO, Tokyo, Japan) were used for collecting IR and CD spectra, respectively. A UV-2102 (Unico, Shanghai, China) instrument and a 241 polarimeter (PerkinElmer, Waltham, USA) were used for measuring the UV spectra and optical rotations, respectively. In addition, the NMR spectra were recorded on the Bruker AVANCE III 500 MHz (Bruker, Billerica, USA). The chemical shifts were measured as *δ* values using CD_3_OD signals as reference (*δ*_H_/*δ*_C_ 3.31/49.0). The UPLC-Q-TOF-MS/MS (Waters xevo G2-s QTOF, Milford, USA) was employed to obtain HR-ESI-MS spectra. A SEP LC-52 instrument (Separation (Beijing) Technology Co., Ltd., Beijing, China) equipped with an MWD UV detector was used to perform semipreparative HPLC, utilizing an ODS column (5 μm, 250 mm × 10 mm, YMC-pack ODS-A, Kyoto, Japan).

### 2.2. Sample Preparation and Extraction for Metabolomics Analysis

#### 2.2.1. Seeds Extract

Three thousand fruit pods of *G. elata* Bl. were collected from Anhui Jinzhai County Shao Biotechnology Co. (Anhui, China). The seeds of *G. elata* Bl. were extracted with 75% EtOH three times, and the solvents were removed under vacuo. The total residue was dissolved in H_2_O and subsequently extracted with EtOAc to obtain the EtOAc extract (3.0 g). The dried extract was resolvent in the 75% EtOH, and then the sample was filtered with a 0.22 μm microporous membrane before UPLC-MS/MS analysis.

#### 2.2.2. UPLC Conditions

The extracts from *G. elata* Bl. seeds were analyzed using a Tandem mass spectrometry system (https://sciex.com.cn/) and UPLC-ESI-MS/MS system (UPLC, ExionLC™ AD, Wuan, China, https://sciex.com.cn/). The UPLC analysis was conducted using an Agilent SB-C18 column (1.8 μm, 2.1 mm × 100 mm) with a mobile phase comprising solvent A (acetonitrile containing 0.1% formic acid, Merck, Shanghai, China, mass pure grade) and solvent B (pure water with 0.1% formic acid, A.S. Watson^TM^ Ltd., Tortola, USA, mass spectrometry purity). The gradient program was as follows (solvent A): 5–95%, 0–9 min; 95%, 9–10 min; from 95 to 5%, 5%, 10–12.5 min. The column oven temperature, flow rate, and injection volume were set at 40 °C, 0.35 mL/min, and 2 μL, respectively. The resulting effluent was directed to an ESI-QTRAP-MS for further analysis.

#### 2.2.3. ESI-Q TRAP-MS/MS

The parameters of ESI were established: ion spray voltages were set to −4500 V (negative modea)/5500 V (positive mode). The collision-activated dissociation (CAD) was high. Source temperature was set to 500 °C. The curtain gas, ion source gases I, and gases II were maintained at 25, 50, and 60 psi, respectively. The declustering potential (DP) and collision energy (CE) for MRM transition were optimized separately. MRM experiments were conducted through QQQ scans with NO as medium collision gas. In the analysis period, corresponding to the metabolites that eluteda, a specific set of MRM transitions was monitored.

#### 2.2.4. Metabolite Identification and Quantification

Due to the rarity of the *G. elata* Bl. seeds involved in this study and the small amounts of the extract, three independent extractions were performed on the samples. The results of these extractions showed minimal differences in their composition. Therefore, one of the samples was selected for primary metabolomics analysis, and others were used in the isolation process. Metabolite identification was based on precise mass, MS^2^ fragments, MS^2^ isotopic distribution, and retention time (RT). The intelligent secondary spectrum matching method, developed in-house, was used to match the secondary spectra and RT of the sample with those in the company’s database. The mass tolerance and MS^2^ tolerance were set to 20 ppm, and the RT tolerance was set to 0.2 min. The metabolites were classified into three levels based on the matching scores:

Level 1: Secondary spectrum and RT matching score > 0.7, indicating high-confidence identification.
Level 2: Matching score between 0.5 and 0.7, indicating medium-confidence identification.
Level 3: Matching of Q1, Q3, RT, DP, and CE with database substances, indicating low-confidence identification.

To ensure the reliability and consistency of the data, strict quality control was implemented throughout the analysis process. Although only one sample was used for primary metabolomics analysis, the consistency of results across repeated extractions ensured the reliability and representativeness of the data.

#### 2.2.5. UPLC-Q-TOF-MS/MS Parameters for Secondary Metabolites Analysis

The *G. elata* Bl. seeds extract was analyzed on a UPLC-MS/MS instrument with a xevo G2-s QTOF system (Waters, Milford, USA) controlled by MassLynx 4.1 software. Waters acuity UPLC-PDA was performed as a chromatographic analysis system. A C-18 column (1.7 μm, 2.1 mm × 100 mm, Waters, Milford, USA) was employed for the analysis. UV absorbance range (200–400 nm) and the column temperature (40 °C) were set. The gradient parameters of mobile phase (solvent A: ACN with 0.1% formic acid; solvent B: 0.1% formic acid in water) were set as follows (A): 15%, 0–1 min; 15–23%, 1–15 min; 23–25%, 15–19 min; 25–30%, 19–27 min; 30%, 27–32 min; 30–60%, 32–35 min; 60–80%, 35–43 min, 0.3 mL/min. The lock mass was leucine-enkephalin (200 ng/mL, [M+H]^+^ = 556.2771). MS/MS analysis system was implemented using FAST DDA mode containing a full MS scan (*m*/*z* 50–1500) in positive ion scan mode. The gas flow rate, desolvation temperature, and source temperature were adjusted to 900 L/h, 450 °C, and 100 °C, respectively. The 50 V sample cone voltage and the 3.0 kV capillary voltage were set. Additionally, the collision energy was configured at 4.0 eV for low-energy scans and varied between 20 to 30 eV for high-energy scans.

### 2.3. Molecular Networking

The format of MS/MS data was converted from .raw to .msp and .csv by Progenesis QI, and then, it was transferred to MassIVE (https://massive.ucsd.edu), URL (accessed on 26 November 2024). Molecular networking was made on the GNPS platform. The parameters details were as follows: mass tolerance of precursor ion and fragment ion were set at *m*/*z* 0.02 Da. Molecular networks were assembled using two minimum cluster sizes, five minimum matched fragment ions, and a cosine score of 0.65. The Network Topk was set at 10 and 10 minimum matched peaks should be met in the design formulas. Finally, molecular networking data were visualized in the Cytoscape version 3.9.0.

### 2.4. Purification of Compounds

With a stepwise gradient of MeOH–H_2_O (10–100%), the EtOAc extract (3.0 g) was loaded on the ODS column to afford five major fractions (Fr.1–Fr.10). Four fractions (Fr.4.1–Fr.4.4) were separated from Fr.4 (215.0 mg) by Sephadex LH-20 (MeOH). Two compounds, **3** (*t*_R_ = 30.4 min, 2.4 mg) and **4** (*t*_R_ = 32.4 min, 2.3 mg), were further purified by semi-preparative HPLC (27% ACN-H_2_O) from Fr.4.3. Fr.5 (250.7 mg) was first separated by Sephadex LH-20 (MeOH). The major sample was preliminarily conducted by semipreparative HPLC (33% acetonitrile in H_2_O for 2 min and followed by 33–47% for 20 min, 5 mL/min) to get three subsections (Fr.5.1–Fr.5.3). By semi-preparative HPLC (45% MeOH in H_2_O for 48 min, followed by 45–49% for 12 min, and followed by 49% for 25 min), Fr.5.1 (45.6 mg) was purified to obtain **5** (*t*_R_ = 59.1 min, 1.9 mg), **6** (*t*_R_ = 49.1 min, 6.0 mg), **7** (*t*_R_ = 73.0 min, 5.2 mg), **8** (*t*_R_ = 83.8 min, 1.5 mg), and **10** (*t*_R_ = 42.4 min, 3.8 mg). Then, **9** (*t*_R_ = 65.8 min, 5.2 mg) and **2** (*t*_R_ = 63.2 min, 1.6 mg) were isolated from Fr.5.2 (35.0 mg) by semi-preparative HPLC (32% acetonitrile in H_2_O for 48 min, followed by 32–36% for 12 min, and followed by 36% for 10 min). Two fractions (Fr.6.1 and Fr.6.2) were preliminarily separated from Fr. 6 by Sephadex LH-20 (MeOH). Fr.6.1 (15.2 mg) was further separated to obtain **11** (*t*_R_ = 32.0 min, 3.0 mg) by semi-prep HPLC (58% MeOH). Fr.6.2 (11.0 mg) was further eluted by semi-prep HPLC (53–65% MeOH/H_2_O for 30 min, followed by 65% for 30 min) to obtain **12** (*t*_R_ = 40.5 min, 3.0 mg) and **1** (*t*_R_ = 48.7 min, 2.0 mg).

Cannabisin R (**1**): [α${]}_{D}^{25}$ + 2.0 (*c* 0.1, MeOH); UV (MeOH) *λ*_max_ (log *ε*) 200 (4.06), 286 (3.59), 317 (3.58) nm; IR (neat) *ν*_max_ 3381, 2940, 1596, 1514, and 1255 cm^−1^; ^1^H-NMR (500 MHz, CDCl_3_) and ^13^C NMR (125 MHz, CDCl_3_), Table 1; (+)-HR-ESI-MS: *m*/*z* 936.3713 [M+H]^+^ (calcd. for C_54_H_54_N_3_O_12_, 936.3707).

Cannabisin S (**2**): [α${]}_{D}^{25}$ + 2.0 (*c* 0.1, MeOH); UV (MeOH) *λ*_max_ (log *ε*) 200 (3.89), 288 (2.95), 320 (2.97) nm; IR (neat) *ν*_max_ 3374, 2935, 1652, 1598, and 1515 cm^−1^; ^1^H NMR (500 MHz, CDCl_3_) and ^13^C NMR (125 MHz, CDCl_3_), Table 1; (+)-HR-ESI-MS: *m*/*z* 677.2489 [M+Na]^+^ (calcd. for C_37_H_38_N_2_O_9_, 677.2475).

### 2.5. Growth Promoting Activity and Inhibition Activity Test

#### 2.5.1. Growth Promoting Activity Test on the *M. osmundicola*

Some of the primary metabolites and the secondary metabolites (**3** and **4**) were selected for the growth-promoting activity testing. These compounds were solubilized using DMSO (Shanghai Macklin Biochemical Technology Co., Ltd., Shanghai, China, analytical reagent) or dd H_2_O (A.S. Watson™ Ltd., mass spectrometry purity), and then, the structures were added to the medium to the concentrations of 0.1, 1, 10, and 100 μg/mL. Subsequently, the seed germination fungus (*M. osmundicola*) was cultured in these culture media. The growth rates of these fungi were recorded after 7 days.

#### 2.5.2. Growth Inhibition Activity Test on the *F. oxysporum*

The secondary metabolites (**3** and **4**) were tested for the growth inhibition activity test. They were solubilized using DMSO (Shanghai Macklin Biochemical Technology Co., Ltd., analytical reagent), and then, they were added to the medium to the concentrations 0.1, 1, 10, and 100 μg/mL. Subsequently, *F. oxysporum* was cultured in these culture media. The growth rates of these fungi were recorded after 7 days.

#### 2.5.3. Bioinformatic Analysis

Dunn’s multiple comparisons test (*n* = 5) was applied to the analysis of the growth-promoting and antifungal activities of the pure compounds. The non-parametric test was chosen because the data did not meet the assumptions for normality, making parametric tests such as the *t*-test or ANOVA unsuitable. Dunn’s test was applied to control for the error rate in multiple comparisons and ensure the reliability of the statistical results. In addition, all statistical analyses were performed using GraphPad Prism 9.5. Statistical significance was set at *p* < 0.05 (*), *p* < 0.01 (**), *p* < 0.001 (***), *p* < 0.0001 (****), and data are presented as mean ± standard deviation (SD).

### 2.6. Antioxidant Activities Assay

All isolates (**1**–**12**) were tested at the concentrations of 100 μg/mL with the VC as positive controls and then mixed with DPPH^•^ or ABTS^•+^ solution. In addition, compounds **3**–**5**, and 10 were further tested at the concentrations of 400, 200, 100, 50, and 20 μg/mL. The residual DPPH^•^ radical was quantified spectrophotometrically at 405 nm, while the ABTS^•+^ radical was assessed at 515 nm. In this experiment, ethanol was set as the negative control, and VC was set as the positive control [15].

### 2.7. Anti-Inflammatory Activity Test

RAW 264.7 cells were cultured in high-glucose DMEM and then seeded into 24-well plates at a density of 4000 cells/well. All the isolates and the control were added into plates at a concentration of 50 μM, after 24 h. After 24 h, 10 μg/mL of lipopolysaccharide (LPS) was added to plates. After culturing for another 24 h, the content of nitric oxide (NO) in the supernatant was detected [16].

### 2.8. Cytotoxic Activity Test

Lung adenocarcinoma cells (A549), human breast carcinoma cells (Hep G2), and cervical cancer cells (HeLa) were cultured for the cytotoxic activities of the isolated lignanamides (**1**–**12**) by CCK-8 colorimetric method. The pure lignanamides were tested at the concentrations of 100, 50, 25, 12.5, 6.25, 3.125, 1.5625, 0.78125, and 0.3906 μM with the cis-platinum as positive controls (IC_50_ 14 ± 1.24, 12 ± 2.75, 16 ± 1.56 μM, respectively) [17]. The nonlinear regression was used for the calculation of the IC_50_.

## 3. Results and Discussion

### 3.1. Primary Metabolite Analysis Based on Metabolomics

**Figure 1:**
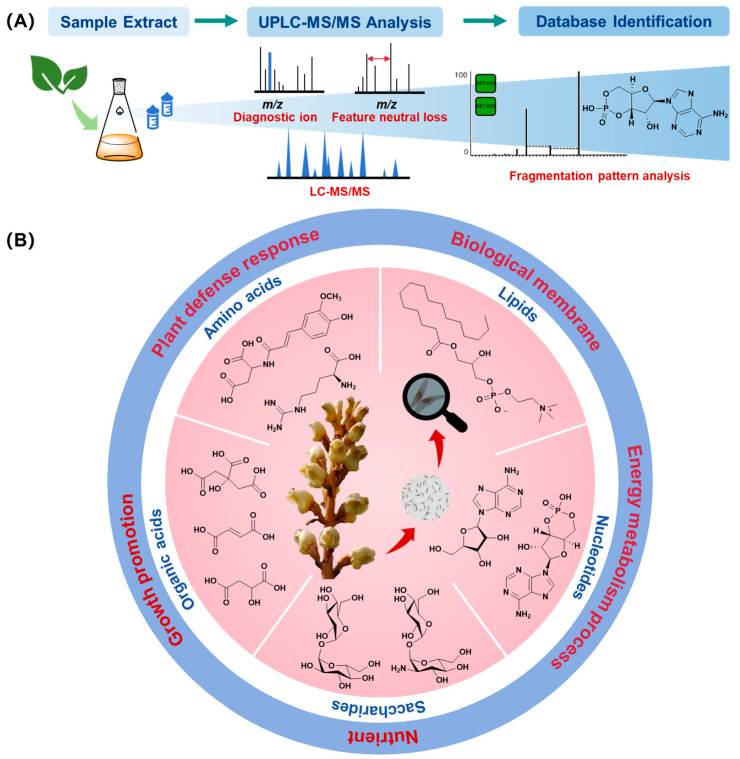
Metabolites analysis process (**A**) and main primary metabolite types from *Gastrodia elata* Bl. seeds (**B**).

Metabolomics analysis of the *G. elata* Bl. seeds was carried out and a total of 1199 primary metabolites were identified. These metabolites were mainly classified into lipids (402), amino acids and their derivatives (359), organic acids (198), carbohydrates (95), nucleotides and their derivatives (120), and vitamins (25) (Figure 1 and Figure S1).

Simple amino acids, dipeptides, and glycoamino acids were the main structure types in the seeds, in which, hydroxythreonine xyloside and *N*-feruloylaspartic acid had the highest percentage. *N*-caffeoylputrescine and *N*′,*N*″-di-caffeoylspermidine were ever reported to play important roles in plant defense responses, considering similar structural features, which indicated that *N*-feruloylaspartic acid may show the defensive function [17]. In addition, Liu reported that the arginine, amino acids histidine, and aspartate acted as chemo-attractants for *Azorhizobium caulinodans*, which showed that these amino acids in the seeds might play a potential role in the establishment of symbiotic relationships between *M. osmundicola* and *G. elata* Bl. seeds [18].

Lipids were mainly classified into five structural types in the seeds: glycerides (53), sphingolipids (44), lysophosphatidylcholines (49), lysophosphatidylethanolamines (51), and free fatty acids (205) (Supporting Data). LysoPG (16:0), phytosphingosine, lysoPC (16:1), and lysoPE (20:3) were the main components in lipids, which involved in the composition of biological membranes and played protective roles for the plants. Cyclic AMP was the major component in the nucleotides and derivatives, which was involved in the energy metabolism process of the plants. These lipids in the *G. elata* Bl. seeds might possess the same biological functions.

Monosaccharides and disaccharides, with small amounts of trisaccharides, were the main components of the *G. elata* Bl. seeds, in which trehalosamine and trehalose accounted for a relatively large proportion. In the symbiotic relationship between orchid plants and fungi, trehalose was considered a common carbon source in fungi, and orchid seeds could utilize trehalose produced by the hydrolysis of fungi as a carbon source to support seed germination [19], whereas trehalose found in the *G. elata* Bl. seeds bring out an interesting question of whether the trehalose in the seed might act as a nutrient constituent through hydrolysis to form glucose for seed germination fungus. Pacheco-Moreno also reported that the glucose and fructose in root exudates could promote the proliferation of beneficial bacteria *Pseudomonas* [20], which implied that trehalose in the seeds might contribute to the symbiotic relationships between *G. elata* Bl. and its seed germination fungus.

Phenolic acids, glycosides, and unsaturated acids were also found in the seeds, in which citric acid accounted for an extremely high proportion. Wen showed that the high release of succinic, citric, fumaric acids, and pyruvic may contribute to the enrichment of *Comamonadaceae* [21], which implied that citric acid in the *G. elata* Bl. seeds might be the potential chemical signal or nutrient element to recruit seed germination fungus or promote its growth.

The content proportion of fat acids, protein, and polysaccharides in the *G. elata* Bl. seeds has been reported through soxhlet extraction and other ways [22]. In this work, the primary metabolites were investigated by the metabolomic method for the first time, which could pave the way for the discovery of potential signaling molecules in the symbiotic relationship between *G. elata* Bl. and its seed germination fungus. Thus, several primary metabolites including aspartic acid, histidine, arginine, glucose, trehalose, fructose, and citric acid were selected for bioactive tests on seed germination fungus *M. osmundicola* and plant pathogen *F. oxysporum*.

### 3.2. Molecular Networking Analysis

**Figure 2:**
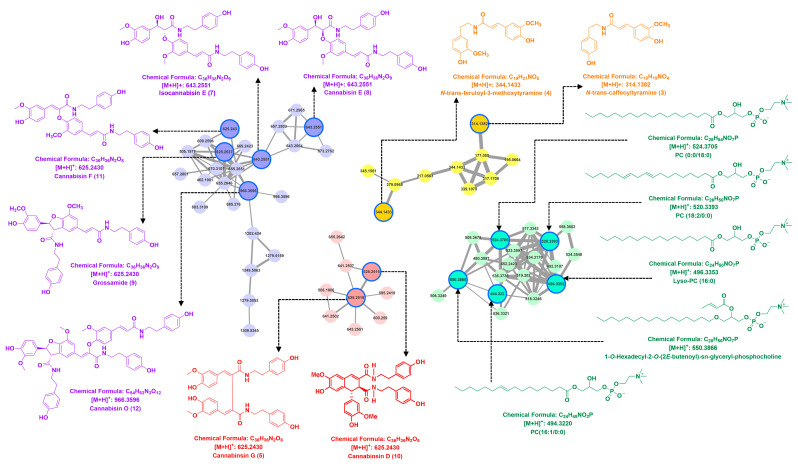
Molecular networking clusters of the seeds of *G. elata* Bl.

To mine secondary metabolites in the seeds of *G. elata* Bl., the crude extract of the seeds of *G. elata* Bl. was examined by the UPLC-Q-TOF-MS/MS. Then, the molecular networking was built by GNPS for the identification and clustering of these secondary metabolites. These structural types were clustered by comparing their MS/MS spectra similarity, from which four clusters (clusters I, II, III, and IV) were well clustered and characterized as lignanamides (clusters I–III), and phospholipids (cluster IV), respectively (Figure 2).

In cluster I, the first lignanamide analog was identified as grossamide (**9**) by the GNPS-embedded library with the consistent molecular formula (C_36_H_36_N_2_O_8_) and MS/MS fragment ion (*m*/*z* 625 and 462). Then, a trimer lignanamide analog canabisin O (**12**) was tentatively identified with the same molecular formula (C_54_H_53_N_3_O_12_) in the literature database [23]. In addition, a pair of stereoisomers canabisin K (**7**) and canabisin E (**8**) were characterized with the same molecular formula (C_36_H_38_N_2_O_9_) and MS/MS fragment ion (*m*/*z* 643, 460, and 314), and the fragment ion at *m*/*z* 460 originated from the loss of the 4-(2-aminoethyl)-phenol unit and a molecular of H_2_O. Structure **11** was speculated as canabisin F with a molecular of H_2_O (18 Da) less than that of canabisin E (isocanabisin E) [24]. Similarly, in the cluster II, two nodes were tentatively characterized as canabisin D (**10**) and G (**5**) with the same molecular formula (C_36_H_36_N_2_O_8_), the common neutral loss of 137 Da ([M+H-C_8_H_10_NO]), and the same MS/MS fragment ions at *m*/*z* 488, *m*/*z* 460, and *m*/*z* 325 as the literature database [24]. In cluster III, two nodes showed the molecular formulas of *m*/*z* 314 and 344, and they showed the identical ion at *m*/*z* 177, which indicated that they could lose the caffeoyltyramine or 3-methoxytyramine group to obtain a trans-feruloyl unit (*m*/*z* 177). Thus, *N*-trans-caffeoyltyramine (**3**) and *N*-trans-feruloyl-3-methoxytyramine (**4**) were tentatively identified based on the same MS/MS fragment pathways and molecular formula as the report [24]. Finally, in cluster IV, five phospholipids were matched with the GNPS library with the common fragment ions at *m*/*z* 184, which matched with the phosphorylcholine unit.

**Figure 3:**
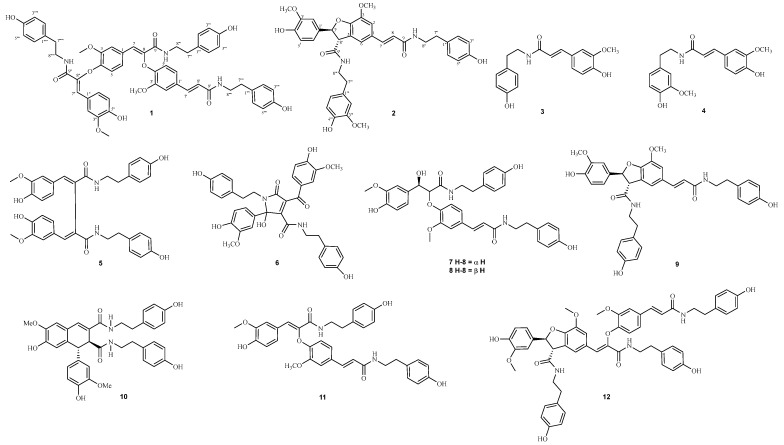
The structure of **1**–**12**.

To verify these preliminarily identified compounds, a systematic separation was carried out on the crude extract of the *G. elata* Bl seeds. Then, 12 lignanamide analogs (**1**–**12**) were isolated, including 10 identified compounds in the molecular network (**3**–**12**) and two unknown analogs (**1** and **2**) (Figure 3). Whereas, phospholipids were not isolated due to the absence of significant ultraviolet absorption, and their structures were finally verified by the reference standards. In addition, the lignanamide analogs (**1**–**12**) were conducted on the antioxidant, anti-inflammatory, and cytotoxicity tests.

### 3.3. Structural Elucidation

**Figure 4:**
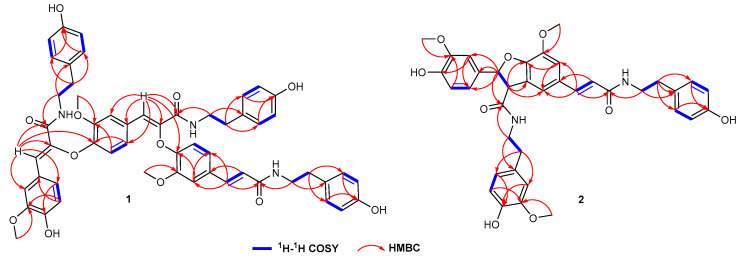
Key ^1^H-^1^H COSY and HMBC correlations of **1** and **2**.

The molecular formula of **1** was determined to be C_54_H_54_N_3_O_12_ based on the HR-ESI-MS (*m*/*z* 936.3713 [M+H]^+^). In the ^1^H-NMR spectra, 12 *ortho*-coupled protons were present at *δ*_H_ 6.59 (H-3‴, 5‴), 6.86 (H-2‴, 6‴), 6.72 (H-3‴, 5‴), 7.06 (H-2‴, 6‴), 6.56 (H-3′′′′′, 5′′′′′), 6.77 (H-2′′′′′, 6′′′′′), which revealed three para-substituted benzene rings (Figure S2). Nine aromatic protons at *δ*_H_ 7.03 (1H, dd, *J* = 8.5, 2.0 Hz, H-6), 6.64 (1H, d, *J* = 8.5 Hz, H-5), and 7.42 (1H, d, *J* = 2.0 Hz, H-2); at 6.96 (1H, dd, *J* = 8.0, 2.0 Hz, H-6′), 6.70 (1H, d, *J* = 8.0 Hz, H-5′), and 7.22 (1H, d, *J* = 2.0 Hz, H-2′); at 6.97 (1H, dd, *J* = 8.0, 2.0 Hz, H-6″), 6.70 (1H, d, *J* = 8.0 Hz, H-5″), and 7.18 (1H, d, *J* = 2.0 Hz, H-2″) suggested the presence of three 1,3,4-trisubstituted benzene rings. Additional, three pair of methylenes *δ*_H_ 3.46 (2H, t, *J* = 7.0 Hz, H-8‴), 2.65 (2H, t, *J* = 7.0 Hz, H-7‴), 3.47 (2H, t, *J* = 7.0 Hz, H-8‴), 2.76 (2H, t, *J* = 7.0 Hz, H-7‴), 3.40 (2H, t, *J* = 7.0 Hz, H-8′′′′′), and 2.56 (2H, t, *J* = 7.0 Hz, H-7′′′′′) suggested the presence of three *N*HCH_2_CH_2_ segments [24]. Combining with a pair of (*E*)-olefinic protons *δ*_H_ 6.47 (1H, d, *J* = 15.5 Hz, H-8′) and 7.44 (1H, d, *J* = 15.5 Hz, H-7′), and two conjugated olefinic protons *δ*_H_ 7.20 (1H, s, H-7″) and *δ*_H_ 7.27 (1H, s, H-7), a rare trimer *N-trans*-caffeoyltyramine was revealed. These three *N*-*trans*-caffeoyltyramine-units were linked by an *O*-atom between C-8″ and C-4, and C-8 and C-4′, which was confirmed by the key *w* long-ranged HMBC correlations from H-7″ to C-4, and from H-7 to C-4′ (Figure 4 and Figures S3–S6). Thus, the planar structure of compound **1** was established. Based on the compound name order in the literature, this compound was named cannabisin R.

**Figure 5:**
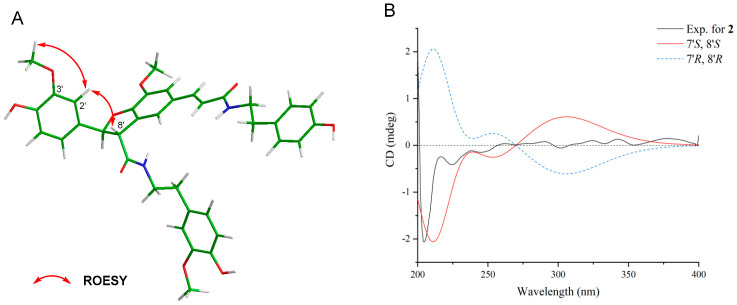
Key ROESY correlations of **2** (**A**) and calculated and experimental ECD spectra of **2** (**B**).

The molecular formula of **2** was determined to be C_37_H_38_N_2_O_9_ based on the HR-ESI-MS (*m*/*z* 677.2489 [M+Na]^+^). In the ^1^H-NMR spectrum, the signals for a *trans*-substituted double bond and a pair of *trans*-oriented oxy-methine appeared as two sets of doublet peaks at *δ*_H_ 7.40 (*J* = 15.5 Hz, H-7) and 6.36 (*J* = 15.5 Hz, H-8′) and at *δ*_H_ 5.88 (*J* = 8.5 Hz, H-7′) and 4.14 (*J* = 8.5 Hz, H-8′), respectively (Figure S7). Four *ortho*-coupled protons were present at *δ*_H_ 6.71 (H-3″, 5″), and 7.07 (H-2″, 6″), which revealed two para-substituted benzene rings. Six aromatic protons at *δ*_H_ 6.75 (1H, dd, J = 8.5, 2.0 Hz, H-6′), 6.79 (1H, d, J = 8.5 Hz, H-5′), and *δ*_H_ 6.91 (1H, d, J = 2.0 Hz, H-2′), and at 6.65 (1H, dd, J = 8.0, 2.0 Hz, H-6‴), 6.74 (1H, d, J = 8.0 Hz, H-5‴), and 6.81 (1H, d, J = 2.0 Hz, H-2‴) suggested the presence of two 1,3,4-trisubstituted benzene rings. Furthermore, two singlets at *δ*_H_ 7.11 (1H, d, *J* = 2.0 Hz, H-6), 6.70 (1H, d, *J* = 2.0 Hz, H-2) indicated a 1,3,4,5-tetrasubstituted benzene ring. Additionally, the presence of two *N*HCH_2_CH_2_ segments was indicated by two pairs of vicinal methylenes *δ*_H_ 2.76 (2H, t, H-7″) and 3.49 (2H, t, H-8″) and 2.82, 2.74 (2H, H-7‴), and 3.59, 3.47 (2H, H-8‴). The HMBC correlations from H-7‴ to C-2‴ and C-6‴, from H-8‴ to C-1‴ and C-9′, and from 3‴-OMe to C-3‴ revealed an *N*-*trans*-feruloyltyramine-like unit. The HMBC correlations from H-7 to C-9, C-6, and C-2, and from H-8 to C-9 and C-1 revealed an *N*-*trans*-caffeoyltyramine-like unit. The correlations from H-8′ to C-5, C-1′, and C-9‴ from H-7′ to C-2′, C-6′, and C-9′ suggested a grossamide-like unit. These data exhibited that compound **2** belonged to the lignanamide family. Except for the presence of an additional methoxy signal (*δ*_H_ 3.82, *δ*_C_ 56.4) in **2**, the signals in ^1^H and ^13^C-NMR of **2** were similar to grossamide [25,26]. This hypothesis was supported by the key HMBC correlation from 3‴-OMe to C-3‴ (Figure 4 and Figures S8–S11). The confirmation of H-8′ on the same face as H-2′ and H-6′ was based on the ROESY correlations from H-8′ to H-2′ (Figure 5A and Figure S12). Thus, the relative configuration of compound **2** was determined. The absolute configuration of **2** was determined by comparing the ECD spectrum recorded in MeOH and the DT-DFT-calculated spectrum of **2** at the B3LYP/6-311+G(2d,p) level [27,28]. The calculated ECD spectrum of **2** matched with the experimental ECD spectrum (Figure 5B), which suggested its stereochemistry to be 7′*S* and 8′*S*. Based on the compound name order in the literature, this compound was named cannabisin S.

### 3.4. Growth-Promoting and Inhibitory Activity of Metabolites

**Figure 6:**
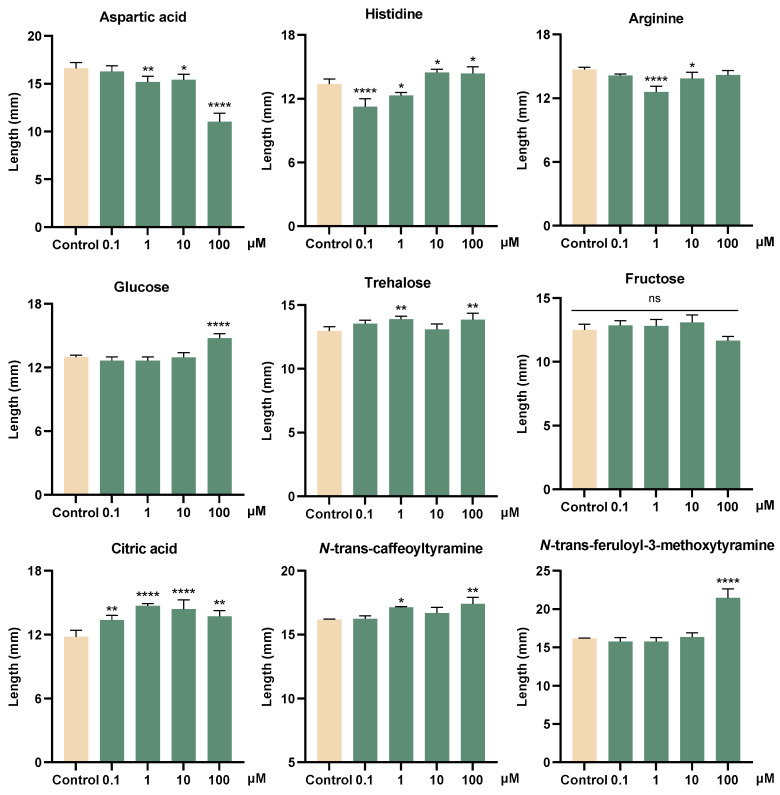
Growth-promoting activity of some metabolites on the fungus *M. osmundicola.* Dunn’s test was utilized to evaluate the statistical significance of the disparity between the treated and control groups, with the corresponding *p* values presented. *p* > 0.05 (ns), *p* < 0.05 (*), *p* < 0.01 (**), *p* < 0.0001 (****).

**Figure 7:**
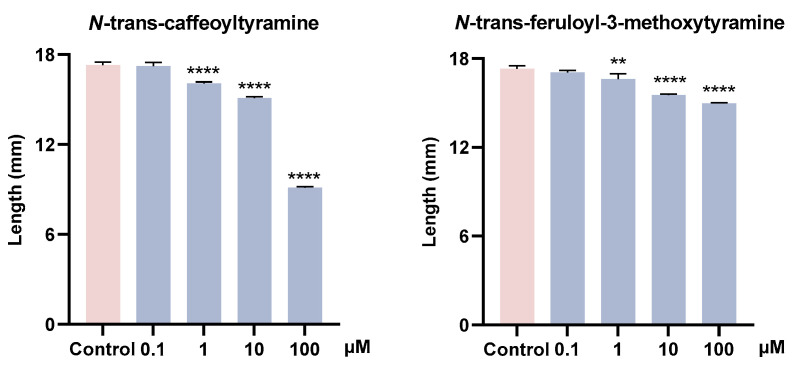
Inhibitory activity of compounds **3** and **4** against *F. oxysporum*. Dunn’s test was utilized to evaluate the statistical significance of the disparity between the treated and control groups, with the corresponding *p* values presented. *p* < 0.01 (**), *p* < 0.0001 (****).

To verify the ecological functions of metabolites in the *G. elata* Bl. seeds, the growth-promoting activities of some primary and secondary metabolites on seed germination fungi (*M. osmundicola*), and their inhibitory activities on plant pathogenic fungi (*F. oxysporum*) were conducted. The experimental results indicated that citric acid promoted mycelial growth at a low concentration (0.1 μM), with its activity showing an initial increase followed by a decline as the concentration increases. Fructose showed no obvious growth-promoting activity, whereas glucose and trehalose could produce active effects at a high concentration (100 μM). Aspartic acid, histidine, and arginine had no obvious growth-promoting effects on *M. osmundicola*, part of which even showed inhibitory effects (Figure 6). Lignanamides analogs (**3** and **4**) showed growth-promoting activities on *M. osmundicola* at high concentrations. In addition, these two compounds also exhibited inhibitory activities on *F. oxysporum* with a concentration-dependent (Figure 7).

These data suggested that at low concentrations, citric acid stimulated mycelial growth and implied that it might act as a potential signal molecule released to the environment by the *G. elata* Bl. seeds to recruit seed germination fungus. Sugars may be utilized as a carbon source by the germination fungus. Although fructose is a common sugar in plant metabolism, it may not be the preferred carbon source utilized by *M. osmundicola* during its growth. In contrast, the growth-promoting effects observed at higher concentrations of glucose and trehalose imply that glucose is more likely the preferred carbon source for *M. osmundicola*, and trehalose may be degraded into glucose and utilized as a carbon source. In addition, lignanamides showed dual ecological functions, promoting the growth of beneficial fungi while inhibiting pathogenic fungi. Therefore, organic acids and lignanamides could be further studied as potential signal/bioactive molecules in the symbiotic relationship between *G. elata* Bl. seeds and its seed germination fungus.

### 3.5. Antioxidant Activity Evaluation

**Figure 8:**
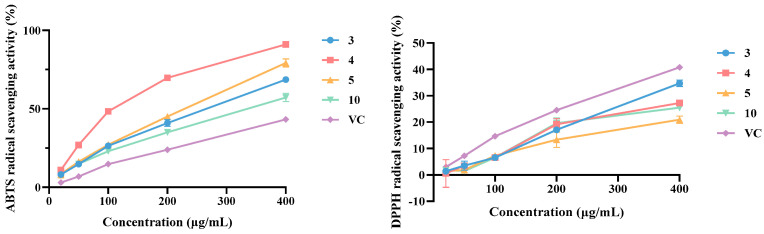
Antioxidant activities of **3**, **4**, **5**, and **10**.

All isolated structures (**1**–**12**) were tested for their antioxidant ability based on DPPH^•^ and ABTS^•+^ radical-scavenging capacity (Table 2). It showed that compounds **1**, **3**–**5**, **7**, **10,** and **11** displayed strong antioxidant activities compared with the positive control VC. Whereas all isolates showed moderate antioxidant activities on the free radical-scavenging capacity toward DPPH. Among all the isolates (**1**–**12**), compounds **3**–**5**, and **10** showed significant antioxidant activities on free radical-scavenging capacity toward ABTS^•+^ than that of the positive control VC (Figure 8). These data suggested that the phenolic hydroxyl groups in lignanamides might be the key active functional group for their antioxidant activity.

### 3.6. Anti-Inflammatory Activity Evaluation

Nitric oxide (NO) plays a significant role in the modulation of various inflammatory diseases as a signaling molecule. This study evaluated the inhibitory effects of all isolated lignanamides (**1**–**12**) on NO production in LPS-activated RAW 264.7 macrophages. The results suggested that all isolates possessed anti-inflammatory effects, with compounds **1** and **9** demonstrating notable inhibition of NO production at 50 μM, showing 35.42 ± 1.24% and 38.21 ± 1.48% inhibition, respectively (Table 3), which suggested they showed similar inflammatory activity potency compared to the positive control dexamethasone sodium acetate.

### 3.7. Cytotoxic Activity Evaluation

To investigate whether the inhibitory activities of these isolates were caused by cytotoxicity, MTT assays were carried out to test their cytotoxic effects on A549, Hela, and MCF-7 cells. The results indicated that none of the compounds had significant cytotoxicity (Table 4). These data were consistent with the previous reports [16,29], which revealed the no/low toxicity of lignanamide analogs, and this class of analogs showed the potential for anti-inflammatory candidate drugs.

Recent research indicated that lignanamides had not been isolated from the tubers of *G. elata* Bl. [14], while these structures were the predominant secondary metabolites in the seeds of this plant. This revealed a significant change in the metabolic products of *G. elata* Bl. during germination, with plants producing metabolites that benefit their specific growth stages. Considering the unique dual biological activities and various pharmacological properties of lignanamides, it is likely that these compounds play an important defensive role for the *G. elata* Bl. seeds, helping them resist adverse environmental conditions. Moreover, since the seeds are not currently used as a medicinal part of the *G. elata* Bl., this work provided new potential for the medicinal value of the *G. elata* Bl. seeds by revealing the pharmacological activities of lignanamide analogs.

## 4. Conclusions

No reports on lignanamide analogs isolate them from the *G. elata* Bl. tuber, indicating that secondary metabolite differences exist in the different parts of the orchid *G. elata* Bl. In addition, all isolates exhibit multiple activities, such as anti-inflammatory and anti-oxidant activities, which might be related to the self-protection of the seeds to resist adverse environments, or these specific molecules might be potential signaling molecules for establishing the symbiotic relationship between *G. elata* Bl. seeds and fungi.

Collectively, primary metabolites and secondary metabolites of *G. elata* Bl. seeds were explored based on metabolomics analysis and UPLC-Q-TOF-MS combined molecular network strategy. A total of 1199 primary metabolites were analyzed from the seed extract, and their metabolites were mainly classified into amino acids, lipids, nucleotides, organic acids, carbohydrates, and vitamins. Of these, citric acid exhibited growth-promoting activity on *M. osmundicola.* Part of the saccharides showed growth-promoting activity at high concentrations, while amino acids did not have obvious growth-promoting activity. A rare class of lignanamide analogs (**1**–**12**) were isolated from the seeds of *G. elata* Bl. These secondary metabolites showed the growth-promoting activity on *M. osmundicola* and an inhibitory effect on *F. oxysporum*, suggesting the dual ecological functions. In addition, since the seeds are not currently used as a medicinal part of the *G. elata* Bl., this work provided new potential for the medicinal value of the *G. elata* Bl. seeds by revealing the strong antioxidant and anti-inflammatory activities of lignanamide analogs.

## Figures and Tables

**Table 1:** ^1^H and ^13^C NMR data of compounds **1** and **2** in CD_3_OD.

Position	1		2	
	*δ*_H_ ^a^ (Mult, *J* in Hz)	*δ*_C_ ^b^, Type	*δ*_H_ ^a^ (Mult, *J* in Hz)	*δ*_C_ ^b^, Type
1		129.0, C		130.5, C
2	7.42, d, (2.0)	114.2, CH	6.70, d, (2.0)	117.8, CH
3		150.1, C		146.0, C
4		147.2, C		151.2, C
5	6.64, d, (8.5)	114.6, CH		129.4, C
6	7.03, dd, (8.5, 2.0)	125.5, CH	7.11, d, (2.0)	113.4, CH
7	7.27, s	124.3, CH	7.40, d, (15.5)	141.7, CH
8		143.1, C	6.36, d, (15.5)	119.5, CH
9		165.2, C		169.0, C
1′		132.0, C		132.6, C
2′	7.22, d, (2.0)	112.5, CH	6.91, d, (2.0)	110.5, CH
3′		15.0.5, C		149.0, C
4′		147.3, C		148.1, C
5′	6.70, d, (8.0)	115.1, CH	6.79, d, (8.5)	116.3, C
6′	6.96, dd, (8.0, 2.0)	122.3, CH	6.75, dd, (8.5, 2.0)	120.0, C
7′	7.44, d, (15.5)	141.1, CH	5.88, d, (8.5)	90.0, CH
8′	6.47, d, (15.5)	121.1, CH	4.14, d, (8.5)	58.8, CH
9′		168.7, C		172.9, C
1″		125.4, C		131.3, C
2″	7.18, d, (2.0)	113.4, CH	7.07, m	130.8, CH
3″		148.9, C	6.71, m	116.4, CH
4″		149.5, C		157.0, C
5″	6.70, d, (8.0)	116.3, CH	6.71, m	116.4, CH
6″	6.97, dd, (8.0, 2.0)	126.4, CH	7.07, m	130.8, CH
7″	7.20, s	125.3, CH	2.76, t, (7.0)	35.8, CH_2_
8″		141.2, C	3.49, t, (7.0)	42.6, CH_2_
1‴		130.8, C		131.8, C
2‴	6.86, m	130.7, CH	6.81, d, (2.0)	113.4, CH
3‴	6.59, m	116.3, CH		149.3, C
4‴		157.0, C		146.0, C
5‴	6.59, m	116.3, CH	6.74, d, (8.0)	116.4, CH
6‴	6.86, m	130.7, CH	6.65, dd, (8.0, 2.0)	122.4, CH
7‴	2.65, t, (7.0)	35.5, CH_2_	2.82, dt, (14.0, 7.0) 2.74, dt, (14.0, 7.0)	35.7, CH_2_
8‴	3.46, t, (7.0)	42.2, CH_2_	3.59, dt, (14.0, 7.0) 3.47, dt, (14.0, 7.0)	42.0, CH_2_
1‴		131.2, C		
2‴	7.06, m	130.7, CH		
3‴	6.72, m	116.3, CH		
4‴		156.9, C		
5‴	6.72, m	116.3, CH		
6‴	7.06, m	130.7, CH		
7‴	2.76, t, (7.0)	35.8, CH_2_		
8‴	3.47, t, (7.0)	42.6, CH_2_		
1′′′′′		130.8, C		
2′′′′′	6.77, m	130.7, CH		
3′′′′′	6.56, m	116.3, CH		
4′′′′′		156.9, C		
5′′′′′	6.56, m	116.3, CH		
6′′′′′	6.77, m	130.7, CH		
7′′′′′	2.56, (7.0)	35.6, CH_2_		
8′′′′′	3.40, (7.0)	42.2, CH_2_		
3-OCH_3_	3.72, s	56.1, CH_3_	3.89, s	56.8, CH_3_
3′-OCH_3_	3.91, s	56.4, CH_3_	3.76, s	56.3, CH_3_
3″-OCH_3_	3.49, s	55.9, CH_3_		
3‴-OCH_3_			3.82, s	56.4, CH_3_

^a^ Recorded at 500 MHz, ^b^ Recorded at 125 MHz.

**Table 2:** Antioxidant activities of compounds **1**–**12** (percent).

Compound	ABTS (100 μg/mL)	DPPH (100 μg/mL)
**1**	9.22 ± 0.40	6.28 ± 0.83
**2**	19.29 ± 1.13	4.45 ± 0.95
**3**	27.43 ± 1.30	4.90 ± 0.59
**4**	48.49 ± 1.75	8.79 ± 0.89
**5**	27.38 ± 0.80	5.18 ± 0.46
**6**	11.55 ± 0.52	5.34 ± 0.31
**7**	15.05 ± 0.89	5.33 ± 0.63
**8**	14.30 ± 1.69	3.59 ± 0.52
**9**	11.15 ± 0.19	8.17 ± 0.22
**10**	22.04 ± 1.31	6.15 ± 0.76
**11**	16.34 ± 0.63	8.49 ± 0.33
**12**	10.84 ± 0.99	5.60 ± 0.90
VC	14.93 ± 0.36	11.42 ± 0.08

**Table 3:** Anti-inflammatory activity of compounds **1**–**12.**

Compound	NO Inhibition (%)
**1**	35.42 ± 1.24
**2**	28.16 ± 2.56
**3**	21.87 ± 1.64
**4**	25.77 ± 1.09
**5**	17.80 ± 3.25
**6**	9.73 ± 1.54
**7**	23.64 ± 2.41
**8**	14.25 ± 1.41
**9**	38.21 ± 1.48
**10**	14.66 ± 3.22
**11**	11.79 ± 0.07
**12**	12.67 ± 3.44
Dexamethasone sodium acetate	49.01 ± 1.96

**Table 4:** Cytotoxic activity of compounds **1**–**12.**

Compound	IC_50_ (μM)		
	A549	Hela	HepG2
**1**	33 ± 1.63	35 ± 1.34	25 ± 1.34
**2**	49 ± 2.74	43 ± 2.12	36 ± 2.42
**3**	60 ± 2.34	73 ± 1.84	64 ± 3.57
**4**	24 ± 4.75	31 ± 2.92	28 ± 1.58
**5**	25 ± 1.76	25 ± 2.31	31 ± 2.37
**6**	37 ± 1.30	29 ± 2.50	29 ± 3.42
**7**	52 ± 2.36	67 ± 4.58	26 ± 1.65
**8**	44 ± 3.56	37 ± 2.56	33 ± 2.56
**9**	78 ± 2.46	64 ± 1.34	57 ± 2.58
**10**	25 ± 1.75	31 ± 1.34	41 ± 3.76
**11**	54 ± 3.57	47 ± 2.33	65 ± 1.46
**12**	>100	>100	>100
cisplatin	14 ± 1.24	12 ± 2.75	16 ± 1.56

## Data Availability

The original contributions presented in this study are included in the article/Supplementary Material. Further inquiries can be directed to the corresponding author(s).

## Supplementary Materials

The following supporting information can be downloaded at: https://www.mdpi.com/article/10.3390/plants14060916/s1, Figure S1. Structural types of primary metabolites. Figure S2. 1H NMR spectrum (500 MHz) of compound 1 in CD3OD. Figure S3. 13C NMR spectrum (125 MHz) of compound 1 in CD3OD. Figure S4. 1H-1H COSY spectrum (500 MHz) of compound 1 in CD3OD. Figure S5. HSQC spectrum (500 MHz) of compound 1 in CD3OD. Figure S6. HMBC spectrum (500 MHz) of compound 1 in CD3OD. Figure S7. 1H NMR spectrum (500 MHz) of compound 2 in CD3OD. Figure S8. 13C NMR spectrum (125 MHz) of compound 2 in CD3OD. Figure S9. 1H-1H COSY spectrum (500 MHz) of compound 2 in CD3OD. Figure S10. HSQC spectrum (500 MHz) of compound 2 in CD3OD. Figure S11. HMBC spectrum (500 MHz) of compound 2 in CD3OD. Figure S12. ROESY spectrum (500 MHz) of compound 2 in CD3OD. Figure S13. Inhibitory effect of compounds 1–12 on tumor cell A549. Figure S14. Inhibitory effect of compounds 1–12 on tumor cell Hela. Figure S15. Inhibitory effect of compounds 1–12 on tumor cell MCF-7. Figure S16. Inhibitory effect of cisplatin on tumor cell A549, Hela, and MCF-7. Table S1: Primary metabolomics analysis.

## Abbreviations

The following abbreviations are used in this manuscript:

AM	Arbuscular Mycorrhizal
OM	Orchid Mycorrhizal
LCOs	Lipo-Chitooligosaccharides
NHCPRC	National Health Commission of the People’s Republic of China
CAD	Collision-Activated Dissociation
DP	Declustering Potential
CE	Collision Energy
LPS	Lipopolysaccharide
NO	Nitric Oxide
