# Supplementary material for: Bioactive Metabolites from the Dusty Seeds of Gastrodia elata Bl., Based on Metabolomics and UPLC-Q-TOF-MS Combined with Molecular Network Strategy
Source: Plants (Basel). 2025 Mar 14;14(6):916. doi: 10.3390/plants14060916 (PMC11944823; doi:10.3390/plants14060916)
Supplement: Supplementary file 1 [file plants-14-00916-s001.zip › Supporting Information.pdf]

Supporting Information

Bioactive Metabolites from the Dusty Seeds of *Gastrodia elata* Bl., Based on Metabolomics and UPLC-Q-TOF-MS Combined with Molecular Network Strategy

Yan-Duo Wang[†], Li-Wen Zhong[†], Hui-Qi Fang[†], Zhao Liu[†], Peng Wang[‡], Longfei Li[‡], Lin Chen[ª], Gang Ding[†, *]

[†] State Key Laboratory of Bioactive Substance and Function of Natural Medicines, Institute of Medicinal Plant Development, Chinese Academy of Medical Sciences and Peking Union Medical College, Beijing 100193, People's Republic of China.

[‡] College of Pharmacy, Hebei University, Baoding 071002, Hebei, People's Republic of China.

[ª] Comprehensive Utilization of Edible and Medicinal Plant Resources Engineering Technology Research Center, Zhengzhou Key Laboratory of Synthetic Biology of Natural Products, Zhengzhou Key Laboratory of Medicinal Resources Research, Huanghe Science and Technology College, Zhengzhou 450006, People's Republic of China.

[*]Correspondence should be addressed to gding@implad.ac.cn. orcid.org/ 0000-0002-8178-4788

Table of Contents

Figure S1 Structural types of primary metabolites. 3

Figure S2 ^{1}H NMR spectrum (500 MHz) of compound **1** in CD_3OD 3

Figure S3 ^{13}C NMR spectrum (125 MHz) of compound **1** in CD_3OD 4

Figure S4 ^{1}H-^{1}H COSY spectrum (500 MHz) of compound **1** in CD_3OD 4

Figure S5 HSQC spectrum (500 MHz) of compound **1** in CD_3OD 5

Figure S6 HMBC spectrum (500 MHz) of compound **1** in CD_3OD 5

Figure S7 ^{1}H NMR spectrum (500 MHz) of compound **2** in CD_3OD 6

Figure S8 ^{13}C NMR spectrum (125 MHz) of compound **2** in CD_3OD 6

Figure S9 ^{1}H-^{1}H COSY spectrum (500 MHz) of compound **2** in CD_3OD 7

Figure S10 HSQC spectrum (500 MHz) of compound **2** in CD_3OD 7

Figure S11 HMBC spectrum (500 MHz) of compound **2** in CD_3OD 8

Figure S12 ROESY spectrum (500 MHz) of compound **2** in CD_3OD 8

Figure S13 Inhibitory effect of compounds **1-12** on tumor cell A549 9

Figure S14 Inhibitory effect of compounds **1-12** on tumor cell Hela 9

Figure S15 Inhibitory effect of compounds **1-12** on tumor cell MCF-7 10

Figure S16 Inhibitory effect of cisplatin on tumor cell A549, Hela, and MCF-7 10

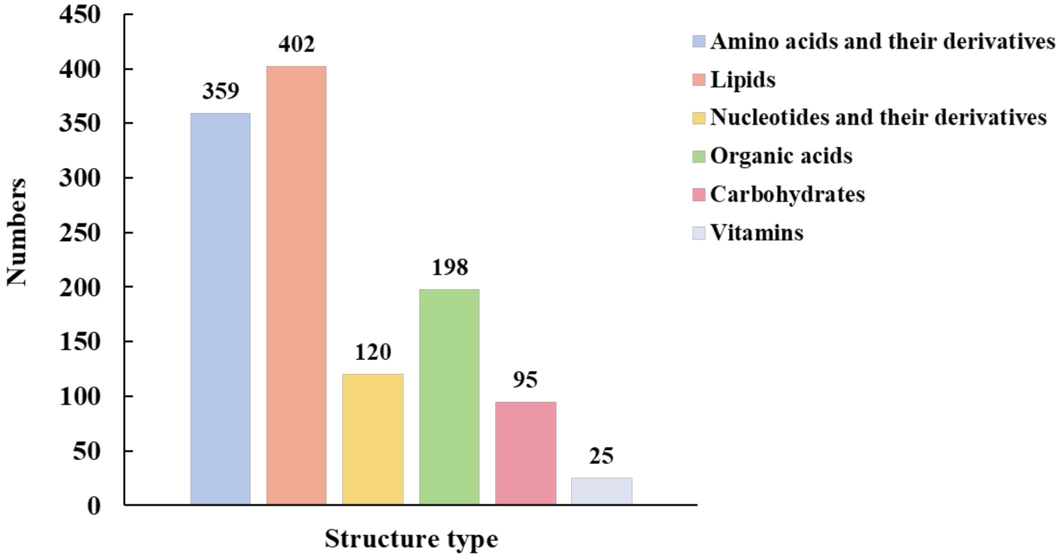

Figure S1 Structural types of primary metabolites.

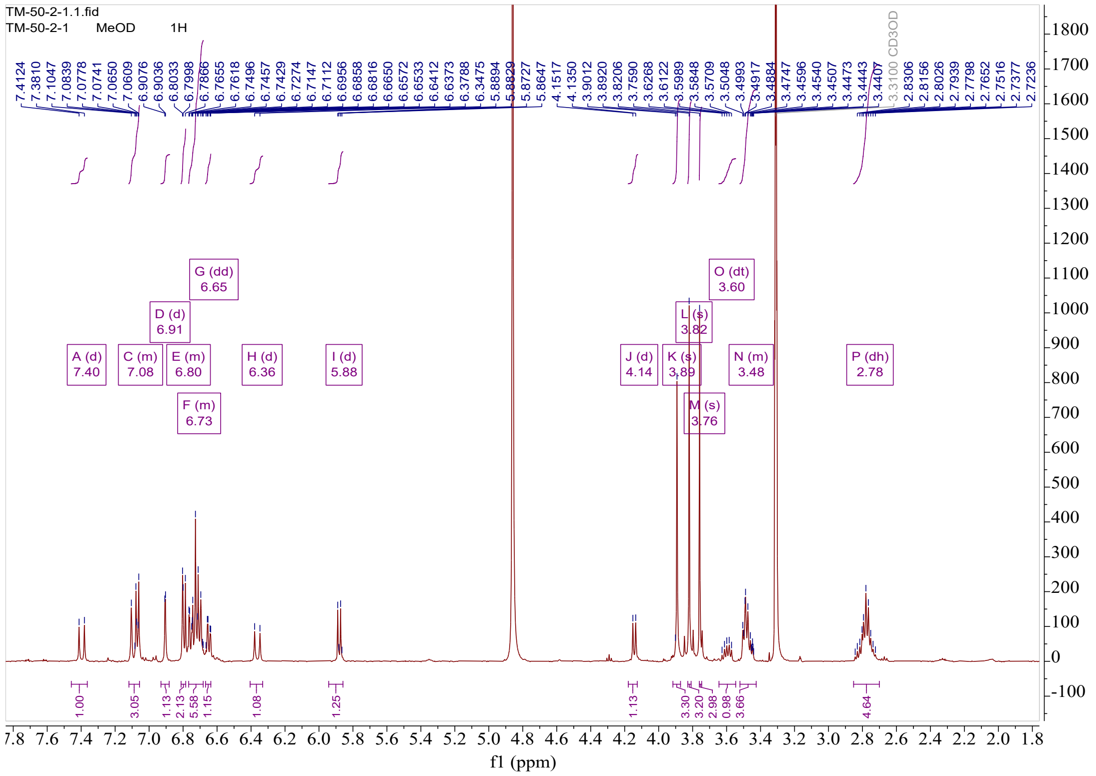

Figure S2 ^{1}H NMR spectrum (500 MHz) of compound **1** in CD_3OD

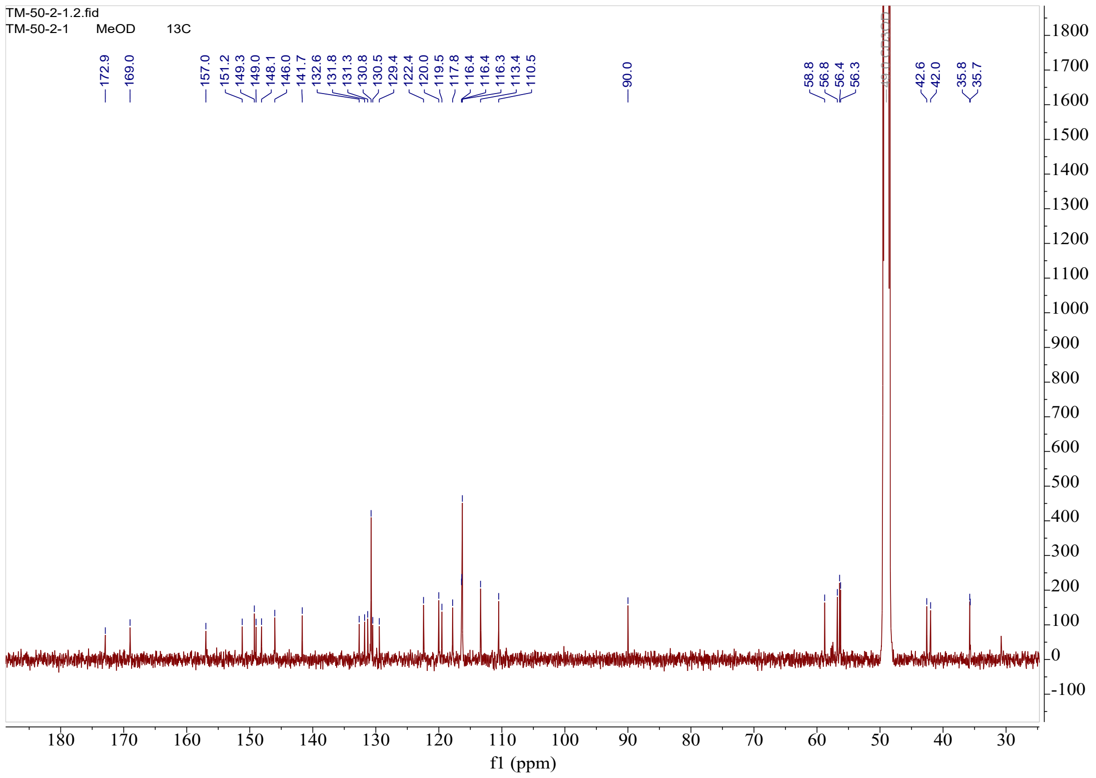

Figure S3 ^{13}C NMR spectrum (125 MHz) of compound **1** in CD_3OD

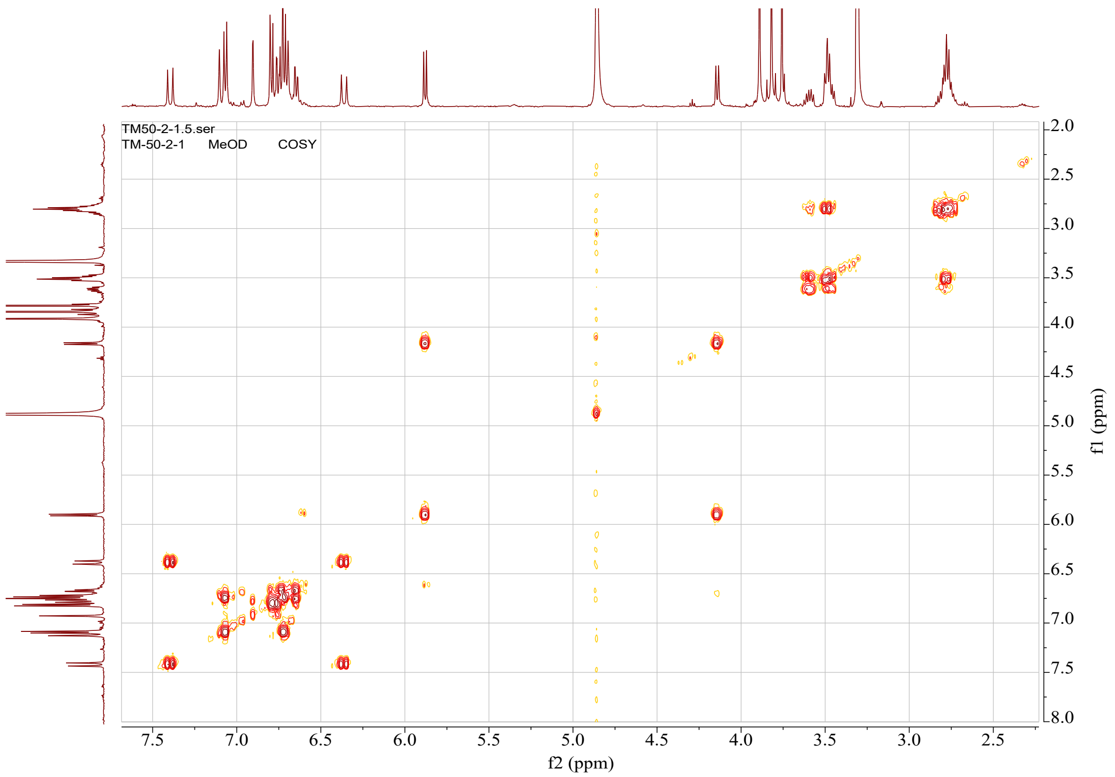

Figure S4 ^{1}H-^{1}H COSY spectrum (500 MHz) of compound **1** in CD_3OD

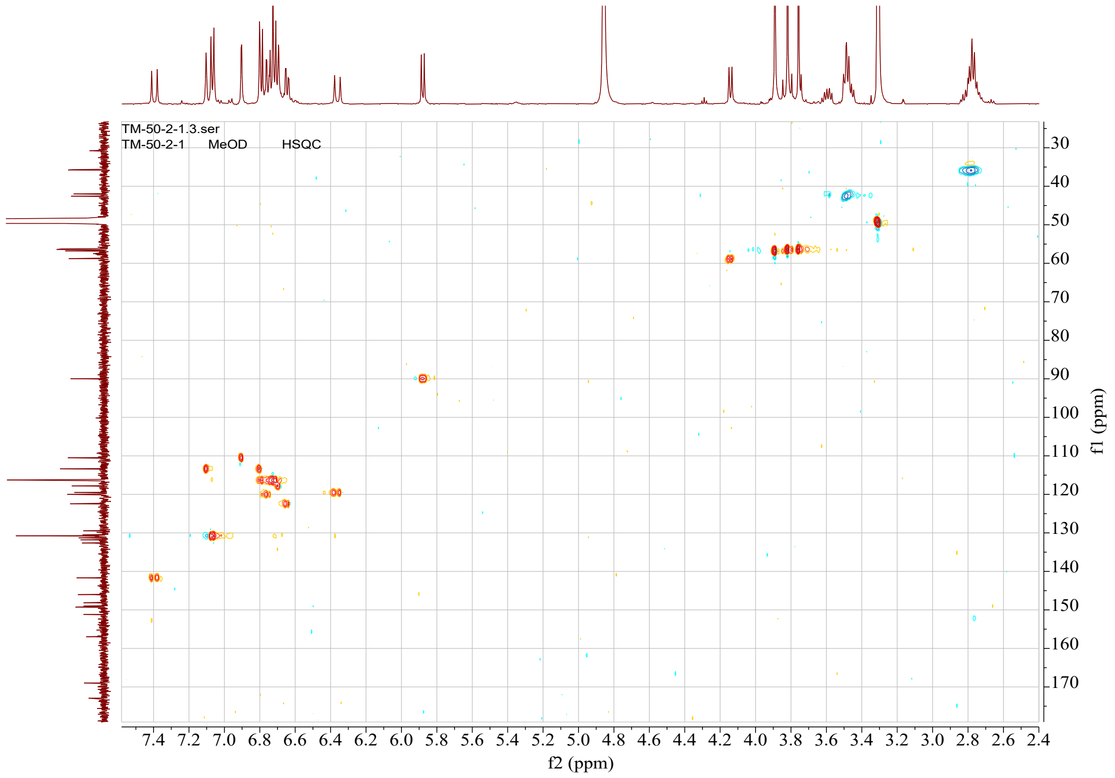

Figure S5 HSQC spectrum (500 MHz) of compound **1** in CD_3OD

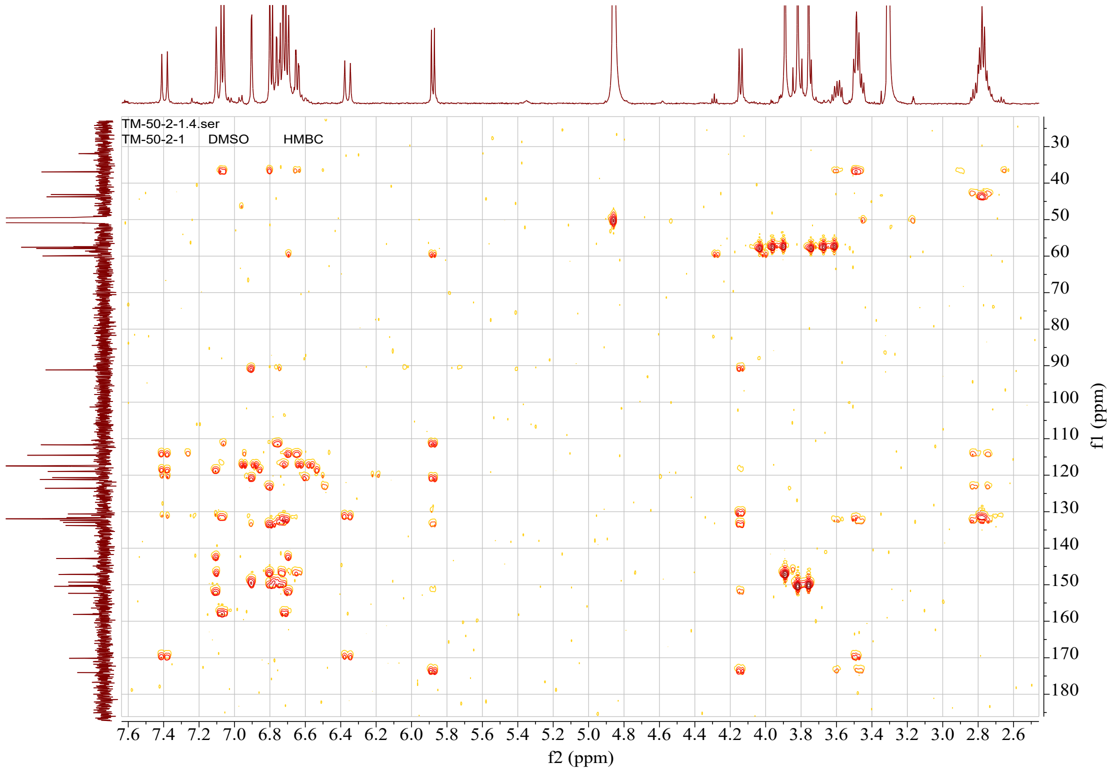

Figure S6 HMBC spectrum (500 MHz) of compound **1** in CD_3OD

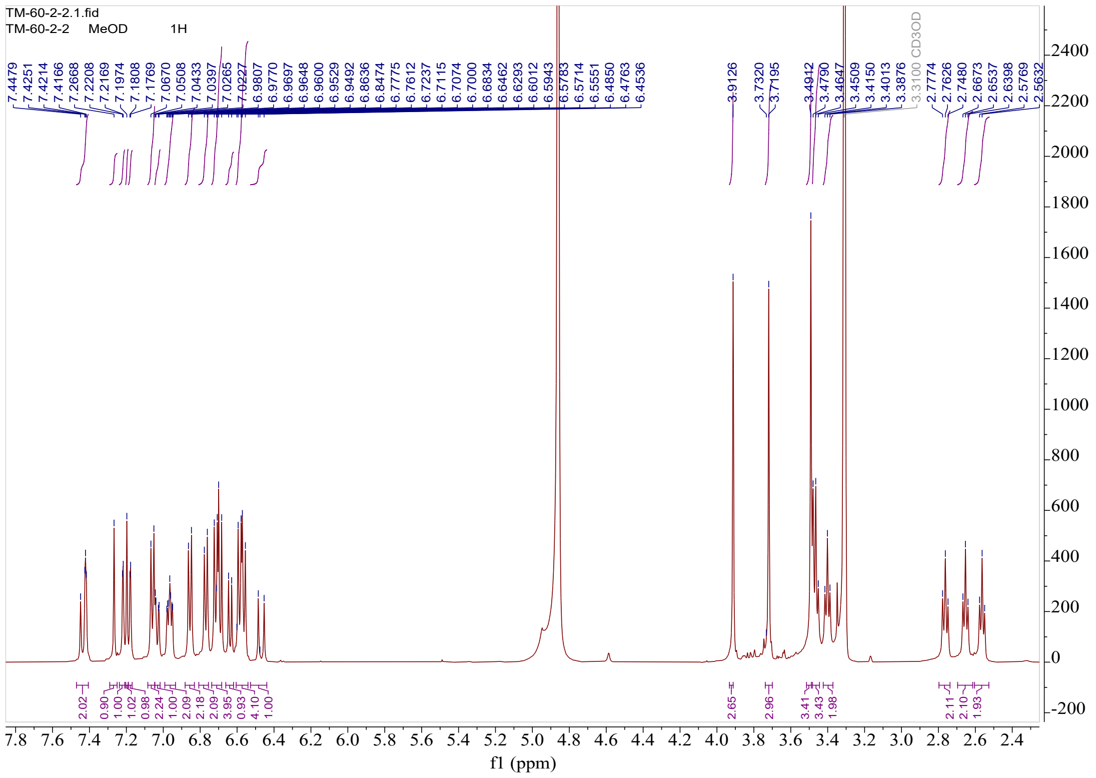

Figure S7 ^{1}H NMR spectrum (500 MHz) of compound **2** in CD$_3$OD

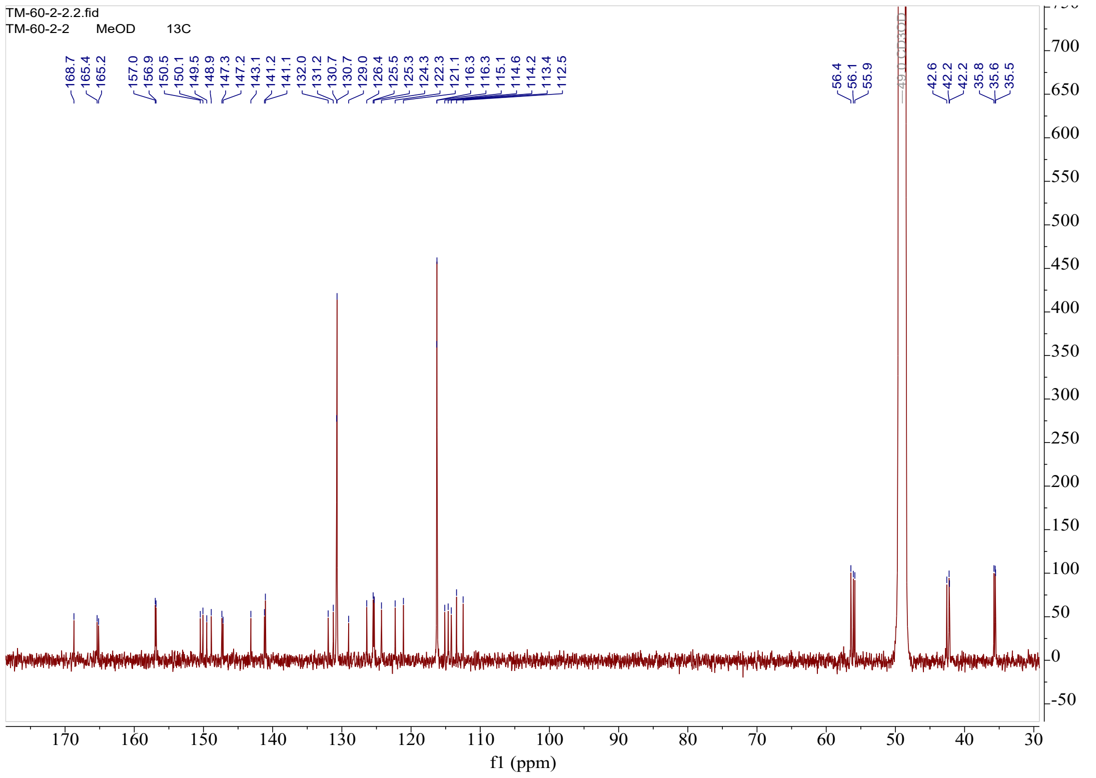

Figure S8 ^{13}C NMR spectrum (125 MHz) of compound **2** in CD$_3$OD

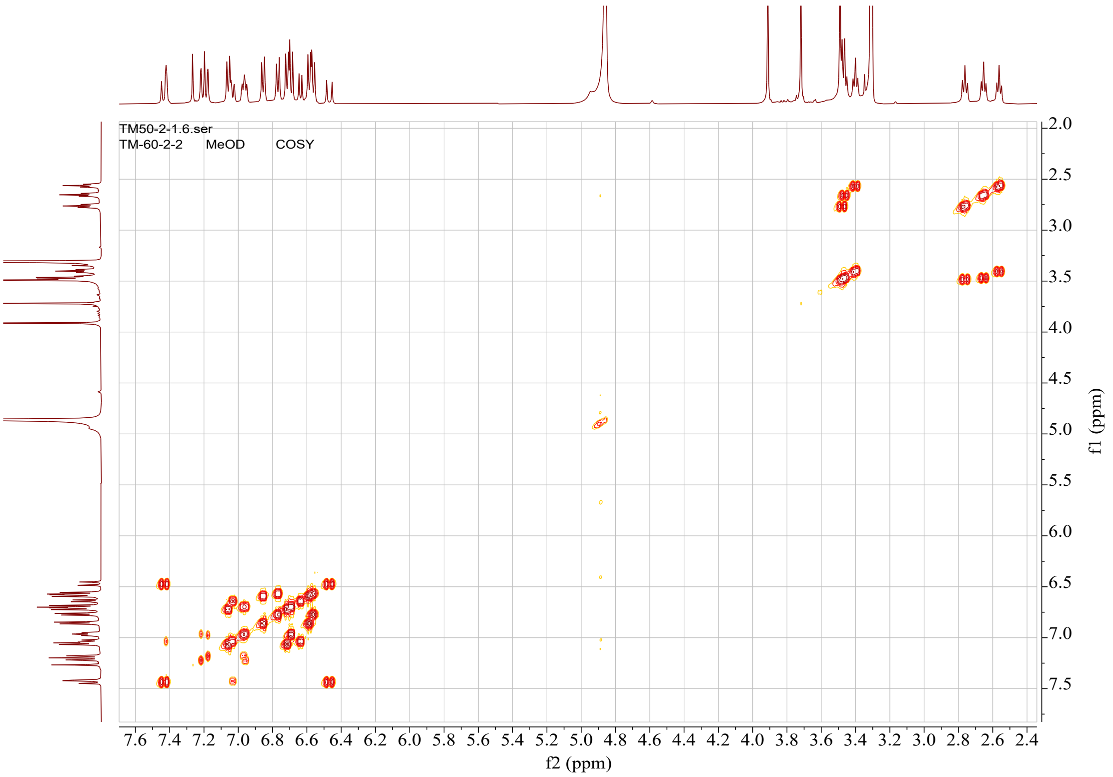

Figure S9 ^{1}H-^{1}H COSY spectrum (500 MHz) of compound **2** in CD_3OD

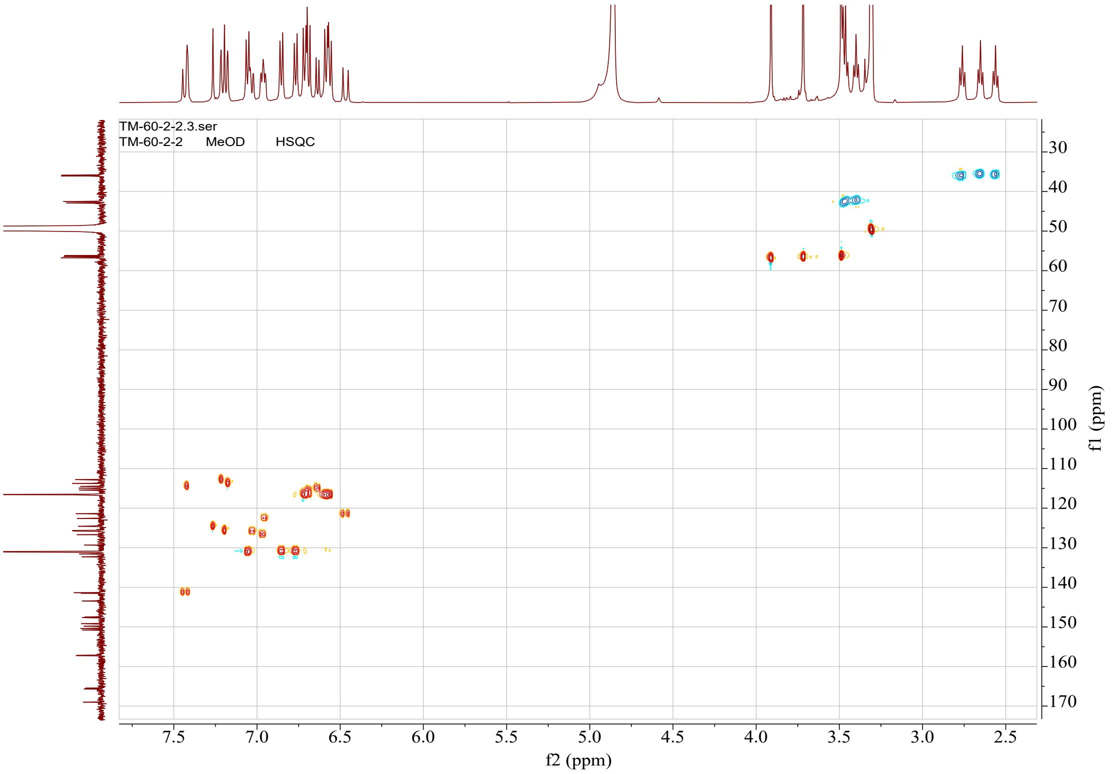

Figure S10 HSQC spectrum (500 MHz) of compound **2** in CD_3OD

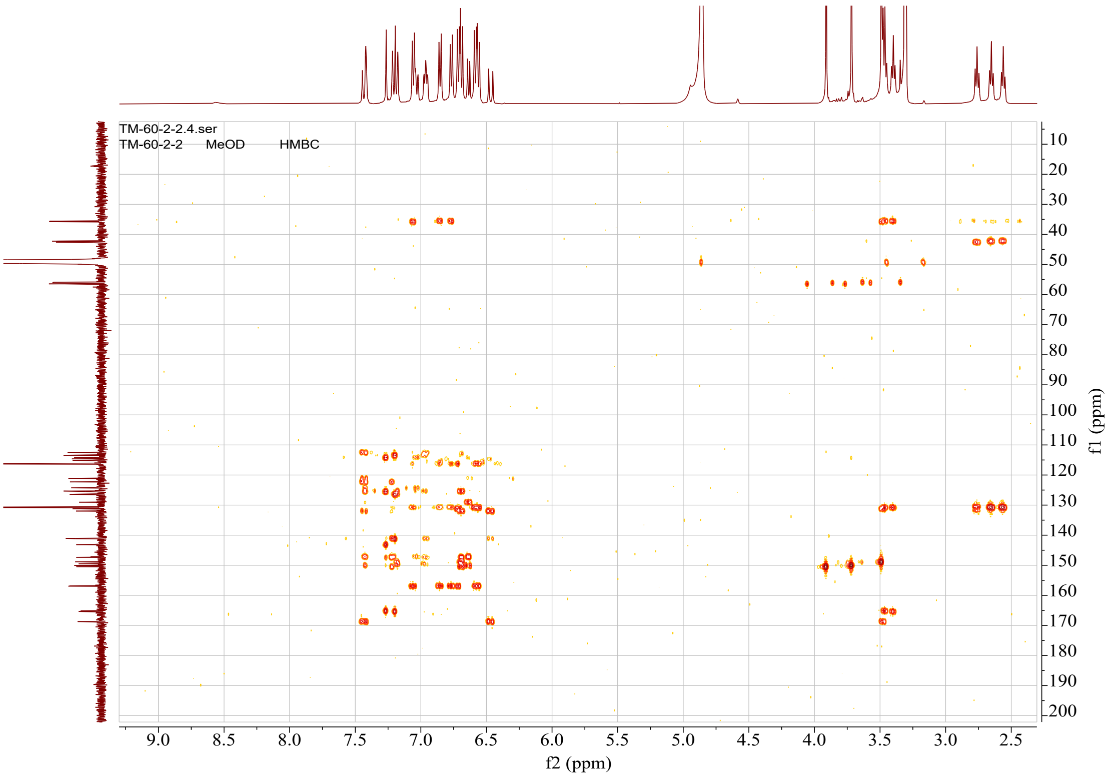

Figure S11 HMBC spectrum (500 MHz) of compound **2** in CD_3OD

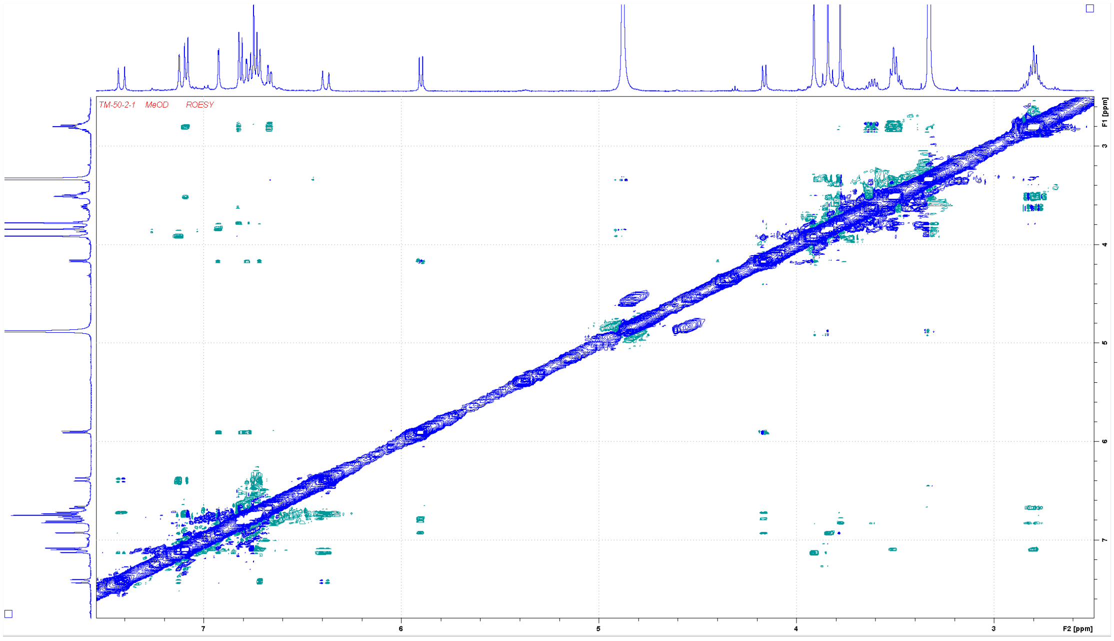

Figure S12 ROESY spectrum (500 MHz) of compound **2** in CD_3OD

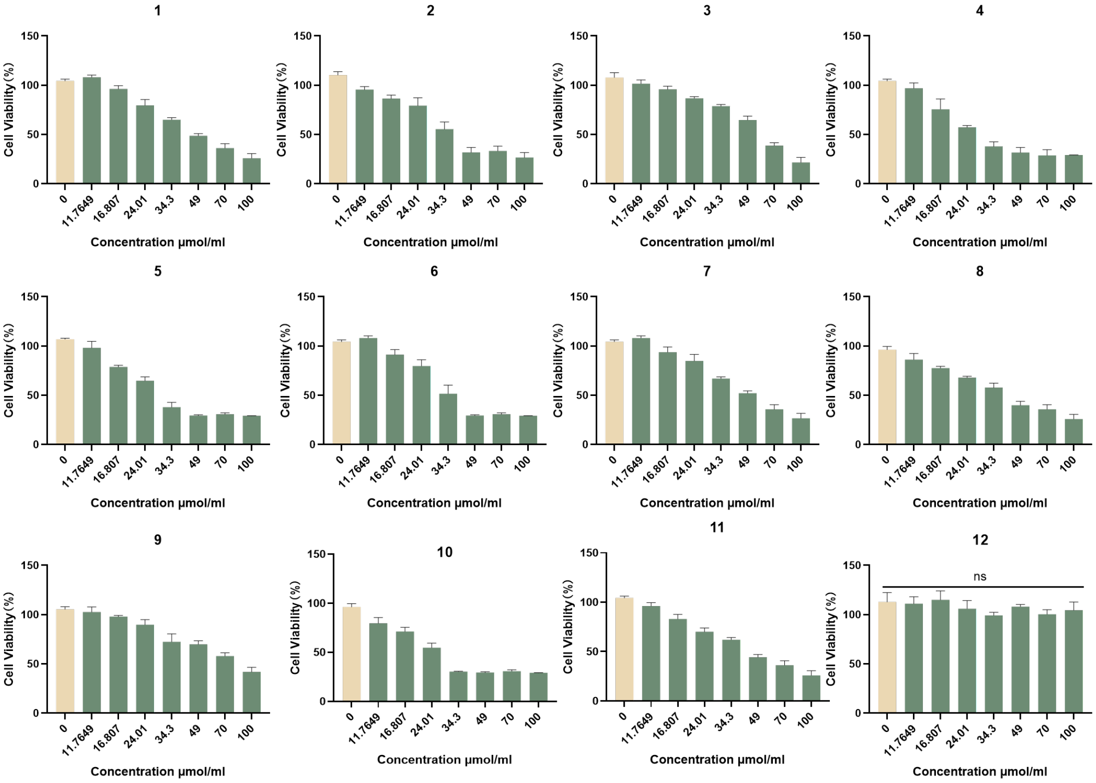

Figure S13 Inhibitory effect of compounds **1-12** on tumor cell A549

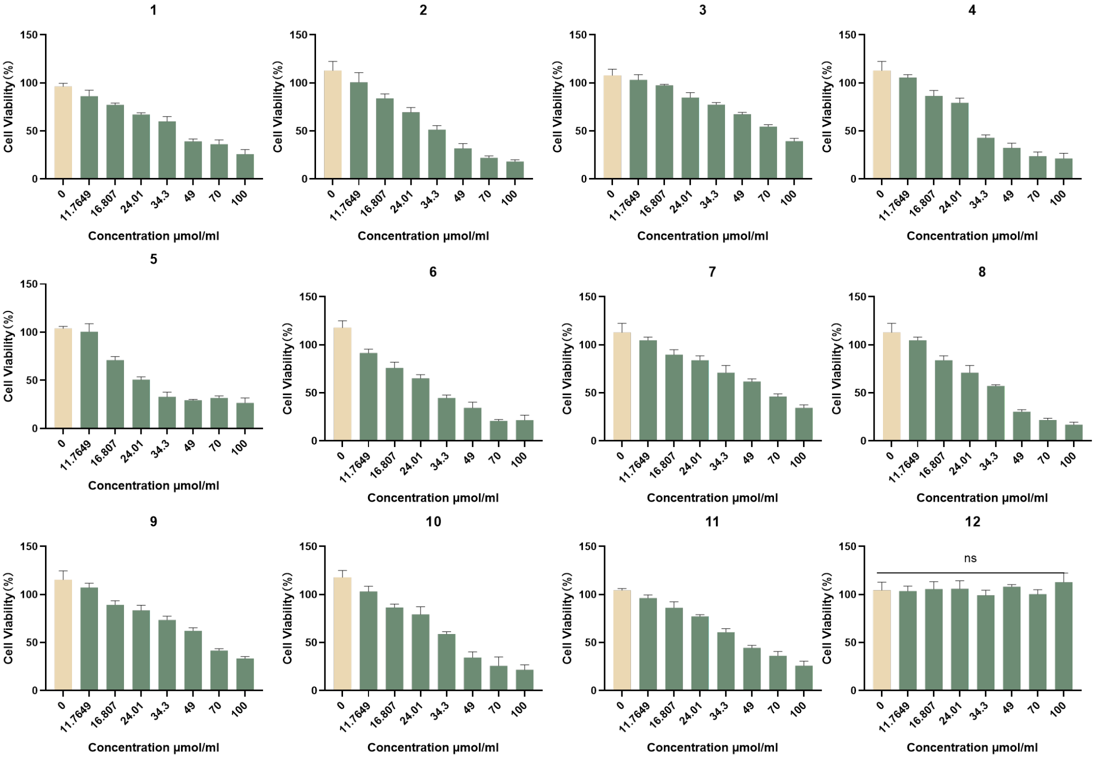

Figure S14 Inhibitory effect of compounds **1-12** on tumor cell Hela

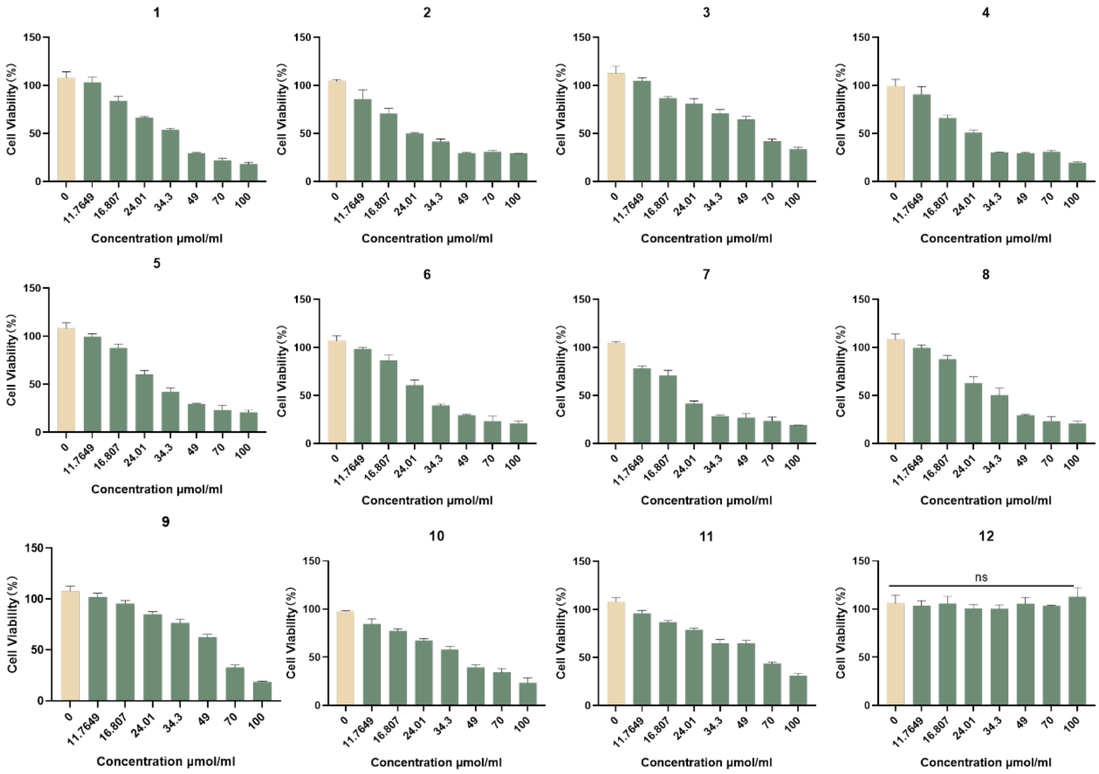

Figure S15 Inhibitory effect of compounds **1-12** on tumor cell MCF-7

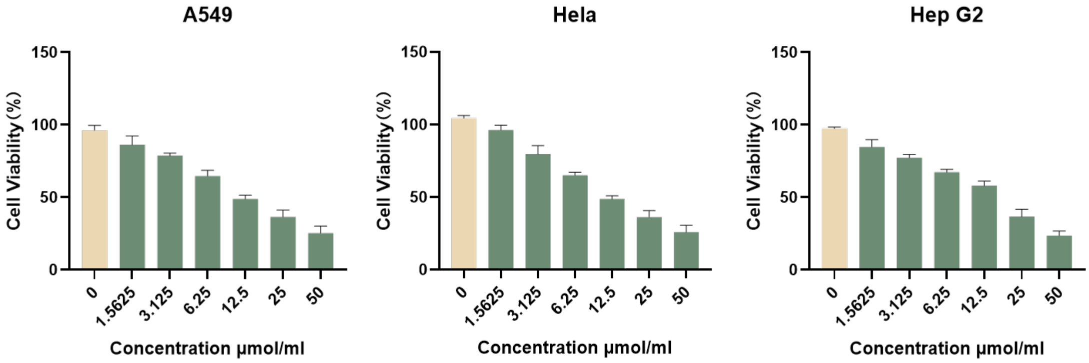

Figure S16 Inhibitory effect of cisplatin on tumor cell A549, Hela, and MCF-7
